# Influence of environmental variables on macroinvertebrate community structure in Lianhuan Lake

**DOI:** 10.1002/ece3.8553

**Published:** 2022-02-14

**Authors:** Qianming Dou, Xue Du, Yanfeng Cong, Le Wang, Chen Zhao, Dan Song, Hui Liu, Tangbin Huo

**Affiliations:** ^1^ Heilongjiang River Fisheries Research Institute Chinese Academy of Fishery Sciences Harbin China; ^2^ College of Fisheries Tianjin Agricultural University Tianjin China; ^3^ Heilongjiang River Basin Fishery Ecological Environment Monitoring Center Ministry of Agriculture and Rural Affairs Harbin China; ^4^ Duerbert Mongolian Autonomous County Fisheries Terminal Daqing China

**Keywords:** community structure, environment variable, Lianhuan Lake, macroinvertebrate

## Abstract

The structural characteristics of the macroinvertebrate community can effectively reflect the health status of lake ecosystems and the quality of the lake ecological environment. It is therefore important to identify the limiting factors of macroinvertebrate community structure for the maintenance of lake ecosystem health. In this study, the community composition of macroinvertebrate assemblages and their relationships with environmental variables were investigated in 13 small lakes within Lianhuan Lake in northern China. A self‐organizing map and *K*‐means clustering analysis grouped the macroinvertebrate communities into five groups, and the indicator species reflected the environmental characteristics of each group. Principal component analysis indicated that the classification of the macroinvertebrate communities was affected by environmental variables. The Kruskal–Wallis test results showed that environmental variables (pH, total phosphorus, nitrate, water temperature, dissolved oxygen, conductivity, permanganate index, and ammonium) had a significant effect on the classification of the macroinvertebrate communities. Redundancy analysis showed that mollusks were significantly negatively correlated with pH and chlorophyll *a*, while annelids and aquatic insects were significantly positively correlated with chlorophyll *a* and dissolved oxygen. Spearman correlation analysis showed that the species richness and Shannon's diversity of macroinvertebrates were significantly negatively correlated with total phosphorus, while the biomass of macroinvertebrates was significantly negatively correlated with pH. High alkalinity and lake eutrophication have a serious impact on the macroinvertebrate community. Human disturbances, such as industrial and agricultural runoff, negatively impact the ecological environment and affect macroinvertebrate community structure. Thus, macroinvertebrate community structure should be improved by enhancing the ecological environment and controlling environmental pollution at a watershed scale.

## Abstract 2

Influence of environmental variables on macroinvertebrate community structure in Lianhuan Lake.

## 1 INTRODUCTION

Lake ecosystems are typical spatially heterogeneous ecosystems formed by interactions between the lake biological communities and their environments. This dynamic process can be illustrated using four‐dimensional changes in biological and environmental elements (Protasov, 2008; Ward, 1989). A lake in a state of natural evolution, undisturbed by humans, possesses a reasonable structure and perfect function; such a lake is in a healthy state (Beck & Hatch, 2009; Cooke et al., 2016). Because of the increase in the human population and the rapid development of industrial production and urbanization, the human demand for water resources has been increasing in recent decades. Problems such as the overexploitation of water resources and environmental pollution have become increasingly prominent (Conor, 2015). As a significant component of the Earth's freshwater resources, lakes also face these problems. The structure and function of lake ecosystems have been seriously damaged, and their health is gradually deteriorating (Likens, 2010; Tilzer & Serruya, 2012).

Macroinvertebrates fulfill various important roles in lakes ecosystems, such as altering the geochemical condition of the sediment, promoting nutrient cycling, and facilitating the transfer of energy within food webs (Cai et al., 2012; Odountan et al., 2019; Vaughn & Hakenkamp, 2001). Macroinvertebrates are also commonly used as indicators of aquatic ecosystem integrity because of their wide range of sensitivity to pollution and relative longevity (Du et al., 2021; Richman & Somers, 2010; Selvanayagam & Abril, 2016). The distribution of macroinvertebrate communities in aquatic systems is strongly affected by both natural factors and human activities (Yu et al., 2020). These natural factors mainly include water temperature, water depth, dissolved oxygen, pH, and the spatial heterogeneity of habitats (Bazzanti et al., 2009; Free et al., 2009; Shostell & Williams, 2007). Human activities, such as agricultural production, indirectly affect the community structure of macroinvertebrates by altering the nutrient levels within lake waterbodies (Wijesiri et al., 2018). Previous studies have shown that there is a strong correlation between macroinvertebrate communities and environmental factors, especially in streams, rivers, and lakes (Kłonowska‐Olejnik & Skalski, 2014; Li et al., 2018; White & Irvine, 2003; Zhang et al., 2021). Thus, analyzing the response relationships between macroinvertebrates and environmental factors has guiding significance for improving the ecological environment of lakes.

Lianhuan Lake, located in Heilongjiang Province, is the largest alkaline lake in northern China. It was formed by a tectonic slump and consists of 18 small lakes (Wu et al., 2012). Lianhuan Lake plays important roles in climate regulation, groundwater replenishment, habitat biodiversity, and economic resource development in northern China (Ma et al., 2018; Wang et al., 2011). However, in recent years, problems such as biodiversity loss and habitat degradation have intensified owing to agricultural reclamation in the lake catchment area and tourism activities within the lake (Li et al., 2009; Sun et al., 2010). Li (2013) showed that Lianhuan Lake has the highest eutrophication level in northeast China. Chen et al. (2020) showed that the zooplankton community in Lianhuan Lake was in the degradation stage. At present, most of the studies conducted in Lianhuan Lake have focused on the assessment of heavy metals and nutrient elements in core sediments (Sun et al., 2010; Xiao et al., 2010).

In this study, the community composition of macroinvertebrate assemblages and their relationships with environmental variables were investigated in 13 small lakes within Lianhuan Lake. The 13 small lakes were chosen because they belong to the same watershed but differ in their natural conditions (e.g., lake morphology and environmental characteristics; see Table 1), which could result in different relationships between their macroinvertebrate assemblages and environmental variables. The aims of this study were (a) to represent the spatial and temporal patterns of macroinvertebrates from 13 lakes in Lianhuan Lake across three seasons, to permit better understanding of the relationships between environmental variables and macroinvertebrates; (b) to identify the indicator taxa characterizing each community group; and (c) to determine the important environmental variables shaping the community structure of macroinvertebrates in Lianhuan Lake. We expected that within‐lake environmental variables would explain a substantial amount of the variation observed in macroinvertebrates, providing a scientific basis for protecting the health and biodiversity of the ecosystems in Lianhuan Lake.

**TABLE 1:** Description of morphometric and environmental variables (mean ± SD) and macroinvertebrates (mean ± SD) from 13 lakes of Lianhuan Lake

Variables	Aobao	Amuta	Beiqin	Delong	Habuta	Huoshaohei	Nashidai	Rbagu	Tiehala	Talahong	Xihulu	Yangcaohao	Yamenqi
Lake morphometrics													
Surface area (km^2^)	18.4	37.4	11.7	7.4	1.4	41.1	11.6	24.8	14.0	52.2	50.6	6.9	19.1
Perimeter (km)	45.9	82.8	20.4	26.1	2.6	57.6	25.6	57.1	39.2	68.9	73.6	27.3	45.5
Environmental variables													
**WD (m)**	3.15 ± 0.06	3.10 ± 0.28	4.00 ± 0.10	2.90 ± 0.09	2.80 ± 0.01	3.23 ± 0.35	2.85 ± 0.05	3.06 ± 0.28	3.10 ± 0.11	2.73 ± 0.40	3.00 ± 0.01	2.90 ± 0.01	2.40 ± 0.01
WT (°C)	19.13 ± 3.16	21.95 ± 0.92	22.10 ± 2.77	21.17 ± 3.00	21.43 ± 2.79	20.53 ± 2.43	21.13 ± 2.62	20.09 ± 2.76	20.82 ± 2.65	20.37 ± 2.78	19.38 ± 1.87	19.77 ± 3.11	19.25 ± 3.32
**DO (mg/L)**	8.65 ± 1.39	8.53 ± 0.30	9.08 ± 0.78	9.12 ± 1.23	8.20 ± 1.04	9.36 ± 0.71	9.32 ± 1.55	9.01 ± 0.52	9.33 ± 0.71	9.81 ± 0.75	8.57 ± 1.77	8.72 ± 1.12	8.26 ± 1.20
**pH**	8.58 ± 0.17	8.78 ± 0.02	8.14 ± 0.10	8.21 ± 0.28	7.81 ± 0.29	8.57 ± 0.12	8.79 ± 0.09	8.48 ± 0.21	8.50 ± 0.17	8.62 ± 0.17	8.68 ± 0.18	8.49 ± 0.21	8.60 ± 0.13
**COND (μS/cm)**	554.08 ± 3.79	586.50 ± 21.92	440.70 ± 22.98	532.82 ± 116.83	783.00 ± 283.44	660.22 ± 107.94	741.67 ± 16.97	647.90 ± 134.38	664.17 ± 116.97	638.45 ± 27.51	756.33 ± 131.67	560.00 ± 7.94	538.70 ± 10.32
**COD_Mn_ (mg/L)**	5.86 ± 0.78	6.53 ± 0.29	6.43 ± 0.46	9.46 ± 3.06	8.57 ± 1.24	6.19 ± 0.95	8.72 ± 4.11	6.10 ± 0.43	7.78 ± 4.51	6.86 ± 0.52	6.16 ± 0.87	7.13 ± 0.37	6.33 ± 0.30
**TP (mg/L)**	0.10 ± 0.01	0.16 ± 0.02	0.06 ± 0.01	0.11 ± 0.02	0.09 ± 0.02	0.12 ± 0.02	0.16 ± 0.04	0.13 ± 0.08	0.13 ± 0.04	0.14 ± 0.02	0.15 ± 0.02	0.14 ± 0.06	0.14 ± 0.02
**TN (mg/L)**	1.88 ± 1.30	2.24 ± 0.13	1.68 ± 0.23	2.05 ± 0.42	1.96 ± 0.43	1.51 ± 0.37	1.39 ± 0.36	1.68 ± 0.37	1.68 ± 0.39	1.60 ± 0.63	1.03 ± 0.35	1.32 ± 0.27	1.66 ± 0.21
**NH_3_–N (mg/L)**	0.50 ± 0.33	0.94 ± 0.07	0.31 ± 0.13	0.60 ± 0.44	0.74 ± 0.67	0.64 ± 0.38	0.96 ± 0.54	0.61 ± 0.36	0.80 ± 0.46	0.88 ± 0.39	1.02 ± 0.52	0.85 ± 0.49	0.49 ± 0.39
**NO_3_–N (mg/L)**	0.13 ± 0.05	0.11 ± 0.04	0.11 ± 0.01	0.13 ± 0.03	0.17 ± 0.01	0.13 ± 0.05	0.15 ± 0.08	0.10 ± 0.04	0.14 ± 0.05	0.12 ± 0.04	0.14 ± 0.07	0.12 ± 0.01	0.14 ± 0.01
**NO_2_–N (mg/L)**	0.07 ± 0.01	0.08 ± 0.01	0.02 ± 0.01	0.05 ± 0.02	0.07 ± 0.06	0.06 ± 0.02	0.10 ± 0.03	0.07 ± 0.04	0.07 ± 0.02	0.10 ± 0.02	0.10 ± 0.01	0.10 ± 0.03	0.07 ± 0.01
**Chla (μg/L)**	11,729.16 ± 2806.38	27,989.89 ± 437.44	584.50 ± 249.58	10,790.53 ± 8225.70	7756.34 ± 7417.86	10,513.54 ± 8122.56	13,350.05 ± 1148.69	9597.12 ± 7388.88	13,021.56 ± 9734.60	11,838.68 ± 9424.08	12,613.44 ± 9412.88	16,046.17 ± 1354.47	13,793.77 ± 7584.95
**SS (mg/L)**	12.50 ± 10.79	28.00 ± 11.31	6.00 ± 3.68	9.33 ± 7.21	6.66 ± 3.79	23.11 ± 8.88	22.17 ± 14.99	15.56 ± 9.05	24.17 ± 17.01	20.00 ± 7.35	20.66 ± 3.64	23.66 ± 9.24	12.00 ± 1.41
Macroinvertebrates													
**Abundance (ind./m^2^)**	728.0 ± 392.0	1056.0 ± 354.2	496.0 ± 206.8	869.3 ± 150.3	1754.7 ± 768.0	736.0 ± 119.7	1013.3 ± 723.9	794.7 ± 274.4	629.3 ± 324.3	394.7 ± 300.6	1296.0 ± 1172.7	160.0 ± 22.6	504.0 ± 216.0
**Biomass (g/m^2^)**	3.4 ± 2.0	15.8 ± 5.3	25.9 ± 10.8	59.4 ± 56.4	156.8 ± 92.5	2.6 ± 0.9	11.6 ± 10.0	18.7 ± 17.9	34.2 ± 33.0	3.7 ± 2.2	4.3 ± 3.1	4.8 ± 4.0	1.6 ± 1.2

Note

The bold environmental variables and macroinvertebrates indicate significant differences (*p* < .05) among lakes based on Kruskal–Wallis test. Environmental variable abbreviations are described in Section 2.

## 2 MATERIALS AND METHODS

### 2.1 Study area

The study was conducted in Lianhuan Lake, which was formed by a tectonic slump. It consists of 18 small lakes in the low‐lying center of the Songnen Plain in eastern China. The lake has a mean depth of 2.14 m, a maximum depth of 4.6 m, and a surface area of 580 km^2^ (Yu et al., 2001). Lianhuan Lake has become the first international waterfowl hunting ground in China since 1985. It lies in a cold temperate zone characterized by a semiarid climate. The average annual precipitation in the Lianhuan Lake catchment is 400 mm, 70% of which occurs during summer (June to August). From March to May, it is generally dry, with frequent dust storms. Apart from direct precipitation, the northern part of the lake also receives water from the Wuyuer and Shuangyang rivers. Rapid economic development around the lake has led to large quantities of wastewater from agricultural, industrial, and domestic sources being released into the lake. In this study, 13 small lakes in Lianhuan Lake were assessed. The 13 lakes included the main lakes in the upper, middle, and lower reaches of Lianhuan Lake, as well as other lakes of different sizes chosen to reflect the impact of environmental variables on macroinvertebrates. The 13 chosen lakes differ in their natural forms and exhibit different characteristics, including their environmental variables and macroinvertebrate distributions (Table 1). Interestingly, these lakes are connected to each other. Among the 13 small lakes covered in this study, Habuta Lake is geographically furthest from the other lakes. Delong Lake, Yangcaohao Lake, and Beiqin Lake are in the upper reaches of Lianhuan Lake, and Durbote County is located east of these lakes (Wang et al., 2019).

### 2.2 Macroinvertebrate sampling

**FIGURE 1:**
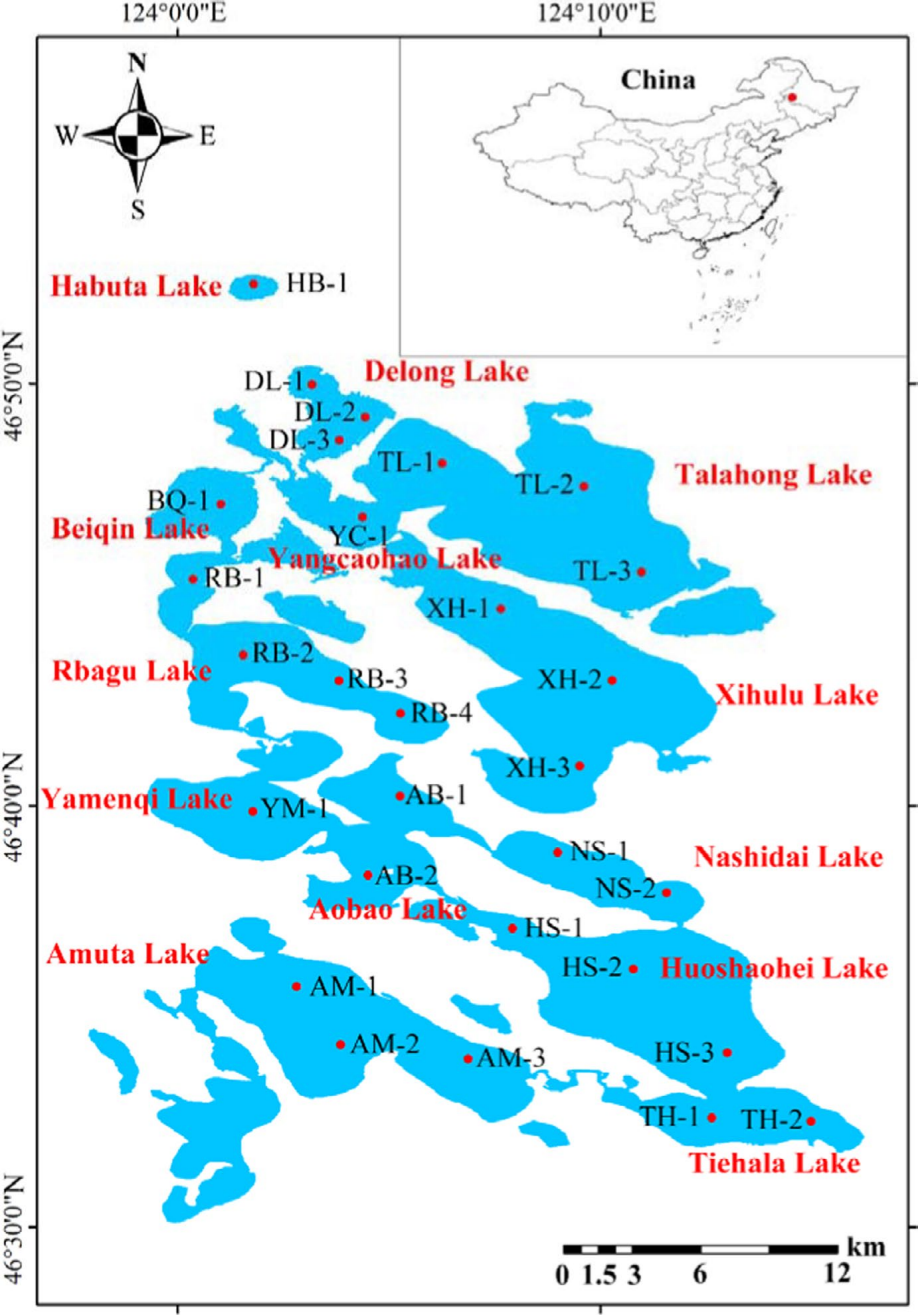
Map showing the locations of the macroinvertebrates sampling sites in Lianhuan Lake

Sampling was conducted at each site in spring (June), summer (August), and autumn (October) of 2020 (Figure 1). The macroinvertebrate samples were collected using a modified Petersen grab (0.0625 m^2^). At each site, replicate quantitative Petersen grab samples were collected and processed through a 500‐μm mesh sieve. The sieve and Petersen grab were visually inspected to ensure that macroinvertebrates adhering to the grab and sieve were transferred to the composite sample. All collected materials were placed in a plastic jar and preserved in 80% alcohol. At the laboratory, all the organisms in each sample were counted, weighed to the nearest 0.1 g, and identified to genus or the lowest possible taxonomic level. The species abundance of each sampling site was calculated as the density (individuals/m^2^), and biomass (g/m^2^) was calculated by adding the biomass of all species. Macroinvertebrates were identified and classified according to Liu et al. (1993), Morse et al. (1994), Tang (2006), and Wang (2002).

### 2.3 Environmental variables

The lake surface areas and perimeters were calculated using ArcGIS (ver. 10.7). At each sampling site, the water temperature (WT, °C), conductivity (COND, μS/cm), pH, and dissolved oxygen (DO, mg/L) were measured in the field using a portable YSI Professional Plus instrument (USA). Water depth (WD, m) was measured using a Speedtech Handheld Depth Finder (USA). Water samples were collected directly using 5‐L polypropylene bottles at a depth of 0.5 m to quantify water chemistry variables. Total phosphorus (TP, mg/L), total nitrogen (TN, mg/L), ammonium (NH_3_–N, mg/L), nitrate (NO_3_–N, mg/L), nitrite (NO_2_–N, mg/L), the permanganate index (COD_Mn_, mg/L), and suspended substances (SS, mg/L) were measured in the laboratory using standard methods (Water Environment Federation & American Water Works Association, 2005). Chlorophyll *a* concentration (Chla, μg/L) was determined according to the protocols for standard observation and measurement in aquatic ecosystems by filtering the water through a GF/C Whatman filter. Pigment extraction was performed in a 90% aqueous solution of acetone, and the Chla concentrations measured spectrometrically.

### 2.4 Classification of macroinvertebrate communities using a self‐organizing map

To classify the macroinvertebrate communities of the sampling sites according to species abundance, a self‐organizing map (SOM) was applied. SOMs are an effective cluster analysis method with high explanatory ability in the study of ecological populations (Giraudel & Lek, 2001). The SOM consisted of two layers of neurons, the input layer and the output layer, connected using connection intensities (weighted connections). Input layers acquire information from a data matrix, while output layers visualize the computational results (Song et al., 2007). In this study, the input layer comprised species abundance data and 74 sample sites. The number of neurons in the output layer was determined in advance, according to $5\times \sqrt{\text{number of sample sites}}\approx 43$ (Park et al., 2003) and the minimum quantization and topographical errors (see Appendix S1). Thus, the optimal number of neurons in the output layer was determined to be 49 (Kohonen, 2001). The SOM output layer had no distinct classification boundaries. *K*‐means clustering analysis was performed on the SOM output layer neurons to classify the sites into different groups. The simple structure index (SSI), which indicates the relative importance of each species in determining the distribution patterns of the samples in the SOM, was then used to determine the optimal number of groups (Park et al., 2006). The larger the SSI value, the higher the clustering quality (Dimitriadou et al., 2002; Park et al., 2006).

Indicator species are closely related to environmental changes and are used by measuring the specificity and fidelity of a species to a certain environmental state (McGeoch et al., 2002). The indicator values (IndVal) of all species in each group were calculated to determine the indicator species in each group. One thousand permutations were performed to assess the significance (*p* < .05) of the IndVal observed for each species (Arimoro & Keke, 2021). The calculation of the IndVal was performed as follows (Dufrêne & Legendre, 1997):

$$A_{\mathrm{ij}}=N{\text{individuals}}_{\mathrm{ij}}/N{\text{individuals}}_{i}$$

$$B_{\mathrm{ij}}=N{\text{sites}}_{\mathrm{ij}}/N{\text{sites}}_{j}$$

$${\text{IndVal}}_{\mathrm{ij}}=A_{\mathrm{ij}}\times B_{\mathrm{ij}}\times 100$$

where IndVal*_ij_* is the IndVal of species *i* in site cluster *j*. *A_ij_* is a measure of specificity; *N*individuals*_ij_* is the mean number of individuals of species *i* across the sites of group *j*, and *N*individuals*_i_* is the sum of the mean number of individuals of species *i* across all groups. *B_ij_* is a measure of fidelity; *N*sites*_ij_* is the number of sites in cluster *j* where species *i* is present, and *N*sites*_j_* is the total number of sites in that cluster. *B_ij_* is high when species *i* is present in all objects of cluster *j*. Indvals greater than 50% were regarded as indicator species. Five macroinvertebrate community indices (species richness, abundance, biomass, Shannon's diversity, and Pielou's evenness) were also calculated.

### 2.5 Statistical analysis

Prior to analysis, the macroinvertebrate abundance data were log‐ and Hellinger‐transformed for SOM and redundancy analysis (RDA), respectively. With the exception of pH, all environmental variables were log‐transformed to satisfy the normality and variance assumptions before performing RDA and principal component analysis (PCA). Before RDA, the gradient lengths were measured using detrended correspondence analysis. As the first gradient length was <4, a linear method was applied. To analyze whether the classification of the macroinvertebrate communities was affected by environmental variables, PCA was conducted to test the variation in environmental variables in each group, and the correlations between the environmental variables were evaluated. The Kruskal–Wallis test was performed to determine the important variables affecting the classification of macroinvertebrate communities. The relationship between environmental variables and macroinvertebrate species composition was evaluated using RDA. Variance inflation factors were used to test for multicollinearity among the environmental variables. Stepwise forward selection (Monte Carlo test with 999 permutations) was used to determine the environmental variables that were significantly correlated with macroinvertebrate species. The statistical significance of the species–environment correlations for each ordination axis was also determined based on 999 Monte Carlo permutations, and the eigenvalues of the first two axes were used to measure their importance (Ter Braak & Verdonschot, 1995). Spearman correlation analysis was used to evaluate the responses of these community indices to the environmental variables. Two‐tailed Student's t tests were used to test for significance (*p* < .05), and the *p*‐values were adjusted using the multiple comparisons test (Benjamini & Hochberg, 1995). SOM was conducted using the ANN Toolbox in MATLAB (ver. R2010b; MathWorks Inc.). Shannon's diversity and Pielou's evenness were calculated using Primer (ver. E‐v5) (Clarke & Gorley, 2001). *K*‐means clustering analysis, RDA, and detrended correspondence analysis were performed using the “vegan” package (Oksanen et al., 2015) in R (version 3.6.3) statistical software (R Core Team, 2019). Indicator values (IndVal), PCA, ANOVA, and Spearman correlation analysis were performed in the “Labdsv” (Roberts, 2019), “ade4” (Dray & Siberchicot, 2020), “agricolae” (de Mendiburu, 2020), and “psych” packages (Revelle, 2020) in R.

## 3 RESULTS

### 3.1 Environmental variables

**FIGURE 2:**
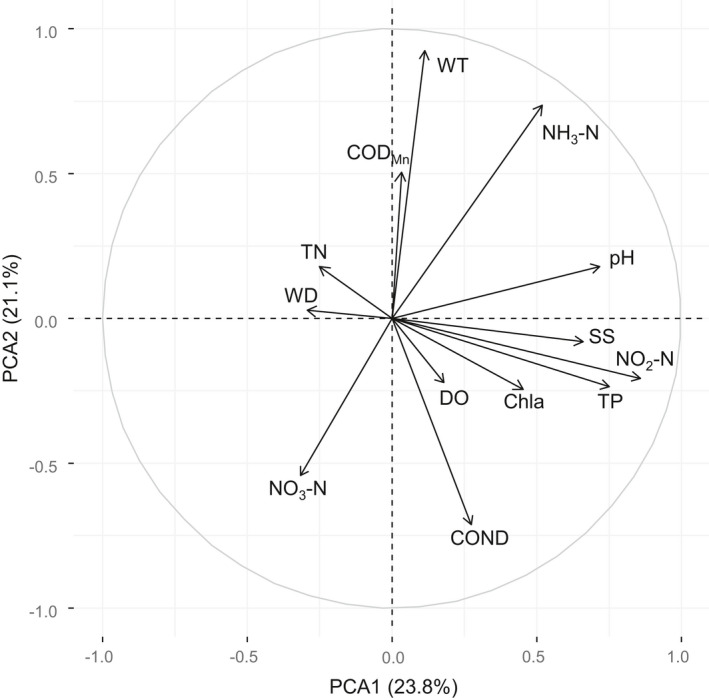
The correlation of environmental variables based on principal component analyses (PCA). Environmental variable abbreviations are described in Section 2

With the exception of WT, the environmental variables showed significant differences (*p* < .05) among the lakes (Table 1). The PCA using 13 environmental variables explained 44.9% of the data variability along the first two axes (axis 1 = 23.8% of the total variance with an eigenvalue of 3.10, axis 2 = 21.1% of the total variance with an eigenvalue of 2.74). Axis 1 was positively correlated with NO_2_–N, TP, pH, SS, Chla, and DO, but negatively correlated with TN and WD. Axis 2 was positively correlated with WT, NH_3_–N, and COD_Mn_, but negatively correlated with NO_3_–N and COND (Figure 2).

### 3.2 Macroinvertebrate community structure

**FIGURE 3:**
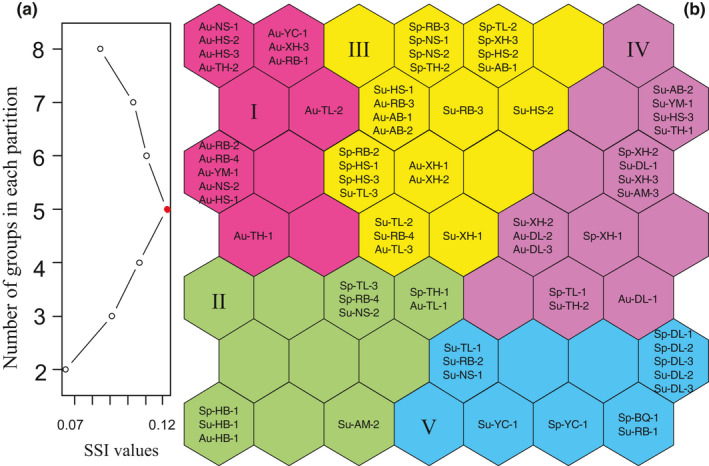
Simple structure index (SSI) and optimal group number (a) Based on SOM neuron *K*‐means cluster analysis. Classification of macroinvertebrate communities (b) Based on Self‐organizing map (SOM). Sp = spring; Su = summer; Au = autumn; I–V = group I–group V

A total of 44 macroinvertebrate taxa (23 aquatic insects, 10 gastropods, 4 bivalves, 4 oligochaetes, 2 leeches, and 1 crustacean) were collected from the 13 lakes in Lianhuan Lake (see Appendix S2). The SSI showed that the clustering quality was the highest when the neurons in the SOM output layer were divided into five groups (Figure 3a). The SOM revealed both spatial and seasonal variation in the classification of the macroinvertebrate communities (Figure 3b). In autumn, most of the sampling sites were grouped into Group I in the top‐left area of the map. Group V, located at the bottom‐right area of the map, mainly included the upper reaches of Lianhuan Lake sampled in spring and summer. In all seasons, the samples from Habuta Lake, which is furthest away from the other lakes, were grouped into Group II in the bottom‐left area of the map. Most sampling sites in the south‐central part of Lianhuan Lake were grouped in Group III in the top area of the map, regardless of season. Most sampling sites in the eastern part of Lianhuan Lake, close to Durbote County, were grouped in Group IV in the top‐right area of the map, regardless of season.

According to the IndVal ≥ 50% criterion, 29 macroinvertebrates were identified as indicator species for the different groups (Table 2). However, 13 species with indicator values lower than 50% (31.89%–48.98%) were also considered significant for particular groups. There was significant variation in the indicator species and number of indicator species among the five groups. Group II had the most diverse indicator species, including one crustacean, three annelids, four mollusks, and five aquatic insects. Group III had only two indicator species, both of which belonged to the family Chironomidae. The indicator species of groups I and IV were dominated by Chironomidae and Mollusca, while the Group V indicator species were mainly characterized by Mollusca. It is worth noting that many of the indicator species, such as *Anatopynia* sp., *Galba pervia*, and *Gyraulus albus*, were distributed into two or more groups (see Appendix S2), implying that the differences in the indicator species between groups mainly resulted from differences in the abundance of taxa in each group.

**TABLE 2:** Indicator values and indicator species of each group of macroinvertebrates in Lianhuan Lake (*p* < .05)

Groups	Indicator species	IndVal (%)	*p*‐Value
I	***Anatopynia* sp**.	**69.15**	.**001**
	***Tanytarsus* sp.2**	**63.19**	.**001**
	***Corbicula fluminea***	**52.80**	.**013**
	***Polypedilum* sp**.	**51.82**	.**001**
	*Chaoborus* sp.	48.98	.001
	*Ephemera* sp.	48.40	.002
	*Conchapelopia* sp.1	41.54	.001
II	***Anotogaster* sp**.	**89.08**	.**001**
	***Glossiphonia* sp**.	**89.08**	.**001**
	***Sphaerium lacustre***	**85.78**	.**001**
	***Sigara* sp.1**	**84.00**	.**001**
	***Sigara* sp.2**	**84.00**	.**001**
	***Valvata piscinalis***	**82.59**	.**001**
	***Mirconecta* sp.1**	**79.40**	.**001**
	***Exopalaemon modestus***	**79.21**	.**001**
	***Branchiura sowerbyi***	**74.60**	.**001**
	***Stenothyra glabra***	**74.48**	.**001**
	***Mirconecta* sp.2**	**73.71**	.**001**
	***Herpobdella* sp**.	**61.31**	.**001**
	***Radix auricularia***	**50.19**	.**004**
	*Parafossarulus striatulus*	44.47	.003
	*Einfeldia* sp.	39.32	.002
III	***Demicryptochironomus* sp**.	**72.29**	.**001**
	***Procladius* sp.2**	**51.95**	.**001**
	*Tanytarsus* sp.1	33.14	.004
IV	***Clinotarypus* sp**.	**79.24**	.**001**
	***Galba pervia***	**58.65**	.**001**
	***Tanypus* sp**.	**52.92**	.**001**
	*Acricotopus* sp.	40.00	.017
	*Limnodrilus* sp.3	37.38	.036
	*Conchapelopia* sp.2	35.83	.004
	*Culicoides* sp.	35.65	.004
	*Procladius* sp.1	31.89	.001
V	***Cricotopus* sp**.	**83.77**	.**001**
	***Radix lagotis***	**83.77**	.**001**
	***Chironomus* sp**.	**67.15**	.**001**
	***Anodonta woodiana***	**62.97**	.**001**
	***Unio douglasiae***	**58.50**	.**001**
	***Gyraulus albus***	**51.76**	.**001**
	***Galaba* sp**.	**50.66**	.**002**
	*Radix ovata*	44.68	.001
	*Radix pereger*	43.50	.002

Note

The bold letters indicate the indicator species for each group having indicator values more than 50%.

### 3.3 Relationship between environmental variables and macroinvertebrate community structure

**FIGURE 4:**
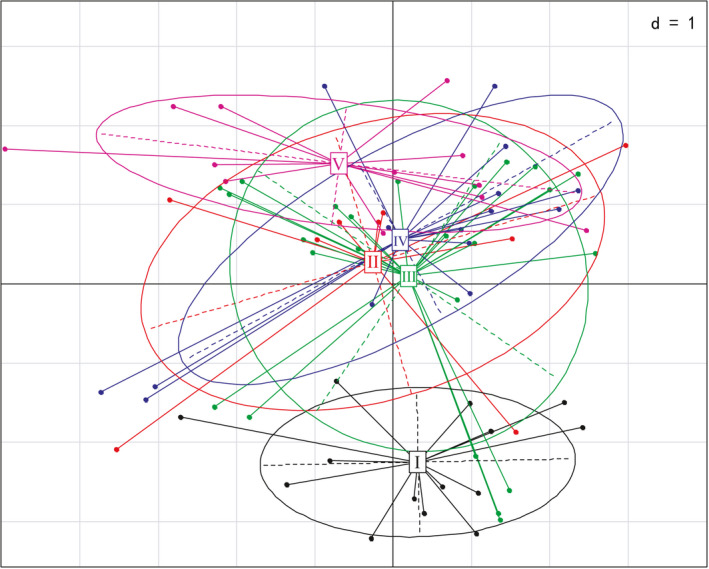
The variation of environmental variables composition among groups based on principal component analyses (PCA). The environmental variables of single groups are represented by ellipses

**FIGURE 5:**
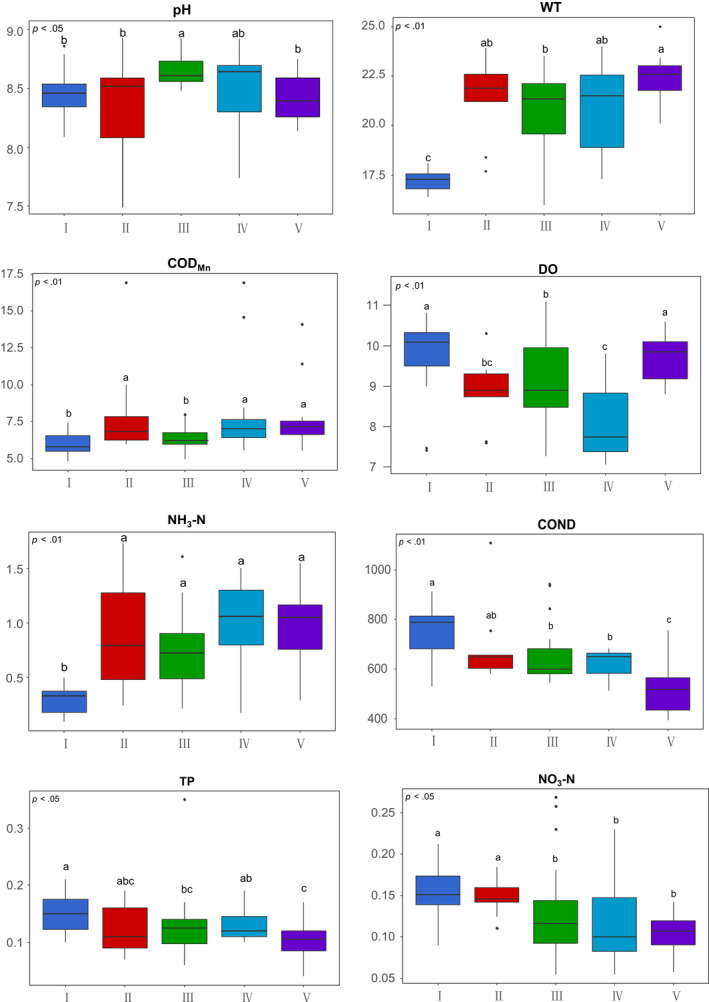
Kruskal–Wallis test boxplot of environmental variable based on self‐organizing map (SOM) grouping. Different letters indicate significant differences (*p* < .05). Environmental variable abbreviations are described in Section 2

Axis 1 of the PCA fundamentally distinguished two groups: Group I and Group V (Figure 4). Kruskal–Wallis test results indicated that pH, TP, NO_3_–N, WT, DO, COND, COD_Mn_, and NH_3_–N had a significant effect on the classification of macroinvertebrate communities (Figure 5). The variation in environmental variables, including WT, COND, pH, DO, TP, NH_3_–N, NO_3_–N, and COD_Mn_, in all seasons across Lianhuan Lake is summarized in Figure 5. All the environmental variables presented were significantly different among the groups. Group I, principally composed of samples collected in autumn, was characterized by low WT (16.40–18.10°C), NH_3_–N (0.09–0.50 mg/L), and COD_Mn_ (4.83–7.45 mg/L) values and high DO (7.41–10.81 mg/L), COND (531.40–931.00 μS/cm), TP (0.10–0.21 mg/L), and NO_3_–N (0.09–0.21 mg/L) values. Group II, which included samples collected in Habuta Lake, was characterized by high COD_Mn_ (5.97–16.92 mg/L), TP (0.07–0.19 mg/L), NH_3_–N (0.24–1.73 mg/L), and NO_3_–N (0.11–0.18 mg/L) values. High pH values (8.48–8.92) and relatively low COD_Mn_ values (4.97–8.00 mg/L) characterized Group III, which comprised sampling sites located in the south‐central part of Lianhuan Lake. The sampling sites located close to Durbote County were grouped into Group IV and were characterized by high COD_Mn_ (5.59–16.92 mg/L), TP (0.10–0.19 mg/L), and NH_3_–N (0.18–1.50 mg/L) values and low DO (7.05–9.80 mg/L) values. Group V, which comprised samples collected in spring and summer from the upper reaches of Lianhuan Lake, was characterized by high WT (20.10–25.00°C) and DO (8.80–10.60 mg/L) values and low TP (0.04–0.17 mg/L), COND (393.90–756.00 μS/cm), and NO_3_–N (0.06–0.14 mg/L) values.

**FIGURE 6:**
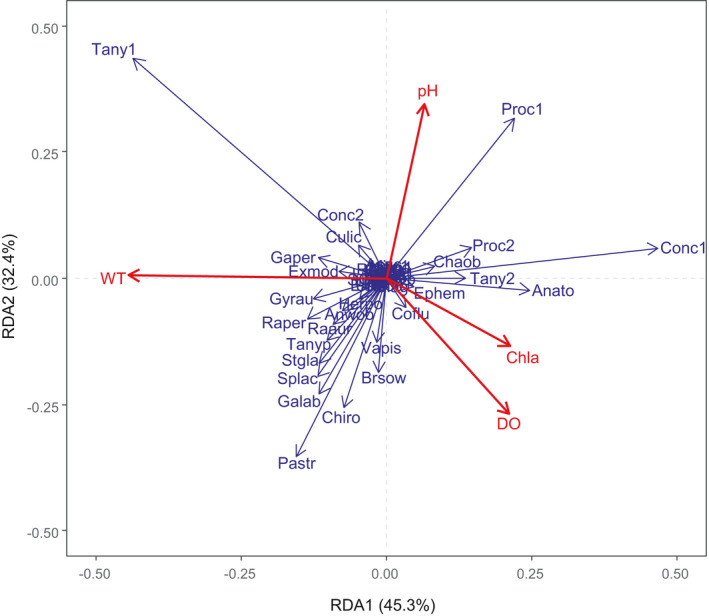
Redundancy analysis (RDA) predicting macroinvertebrate species composition by selected environmental variables. Environmental variable abbreviations are described in Section 2. Macroinvertebrate species abbreviations are described in Appendix S2

The RDA ordination of macroinvertebrate composition with respect to environmental variables is presented in Figure 6. Stepwise forward selection yielded four environmental variables that were significant to the model. These variables were WT, pH, DO, and Chla (Figure 6). These four variables accounted for 77% of the total variance in the macroinvertebrate species composition. The first RDA axis, which explained 45.3% of the total variability, was negatively correlated with WT, whereas the second axis, which explained 32.4% of the variability, was positively correlated with pH. Among the strongest species–environmental associations, we found that mollusks such as *G*. *albus*, *Radix pereger*, and *Stenothyra glabra* were significantly positively correlated with WT and negatively correlated with pH and Chla; annelids such as *Branchiura sowerbyi* and *Herpobdella* sp. were significantly positively correlated with Chla and DO and negatively correlated with pH; and aquatic insects such as *Chaoborus* sp., *Ephemera* sp., and *Anatopynia* sp. were significantly positively correlated with Chla and DO and negatively correlated with WT.

The Spearman correlation analysis (Table 3) showed that macroinvertebrate community indices were significantly affected by environmental variables. Macroinvertebrate abundance was most affected by environmental variables and was significantly negatively correlated with DO (*r* = –.40, *p* = .01), NO_2_–N (*r* = –.33, *p* = .04), and Chla (*r* = –.32, *p* = .04). Both species richness and Shannon diversity were significantly negatively correlated with TP (*r* = –.35, *p* = .04, and *r* = –.34, *p* = .04, respectively). Macroinvertebrate biomass was significantly negatively correlated with pH (*r* = –.39, *p* = .01).

**TABLE 3:** Spearman correlation analysis between macroinvertebrate community indices and environmental variables in the Lianhuan Lake

Environmental variables	Species richness		Abundance		Biomass		Shannon's diversity		Pielou's evenness	
	*r*	*p*	*r*	*p*	*r*	*p*	*r*	*p*	*r*	*p*
WD	–.02	.95	.07	.79	–.04	.85	–.06	.83	–.12	.59
WT	.24	.17	.06	.81	.11	.60	.18	.32	–.05	.83
DO	–.06	.81	**–.40**	.**01**	–.10	.64	.11	.62	.**33**	.**04**
pH	–.07	.81	–.20	.27	**–.39**	.**01**	–.07	.80	.01	.97
COND	–.28	.09	–.04	.85	–.14	.50	–.24	.19	.00	.97
COD_Mn_	.20	.27	.**33**	.**04**	.**42**	.**01**	.04	.85	–.15	.43
TP	**–.35**	.**04**	–.22	.20	–.18	.32	**–.34**	.**04**	–.11	.61
TN	.21	.25	.20	.27	.30	.06	.16	.41	–.05	.83
NH_3_–N	.08	.75	.02	.94	.01	.97	.01	.97	–.13	.51
NO_3_–N	.05	.85	.25	.15	.28	.09	–.04	.85	–.09	.69
NO_2_–N	–.23	.20	**–.33**	.**04**	–.22	.20	–.17	.37	.01	.97
Chla	–.17	.37	**–.32**	.**04**	–.06	.81	–.11	.62	.09	.71
SS	–.14	.50	–.16	.41	–.12	.59	–.03	.92	.19	.29

Note

Data are presented as *r* and *p* values. Significant correlations are highlighted in bold (*p* < .05). Environmental variable abbreviations are described in Section 2.

## 4 DISCUSSION

Understanding the ecological status of lakes helps determine the ecosystem services provided by them (Grizzetti et al., 2016). Therefore, it is important to assess these factors. The ecological status and water quality of a lake are affected by complex interactions between environmental variables. Understanding the relative effects of these environmental variables is a necessary step in determining the activities required for lake management. The macroinvertebrate community is an ideal indicator as they respond to a wide variety of physical, chemical, and biological factors (Rai et al., 2019). This study aimed to investigate the response of macroinvertebrate community structure to environmental variables in Lianhuan Lake.

The spatial patterns of the macroinvertebrate communities were used to classify the lakes for biogeographical division. Based on the species composition, SOM grouped the sampling sites into five groups. This classification implies that spatial variation correlates with macroinvertebrate communities at a relatively small scale. The Kruskal–Wallis test indicated that the environmental variables differed significantly among the five groups. Groups with similar macroinvertebrate communities were placed close to each other in the SOM. Groups I and V were placed furthest from each other, and this was further depicted in the final PCA results.

Group I, primarily composed of samples collected in autumn, was characterized by low WTs and high DO, COND, TP, and NO_3_–N values. The high DO, COND, TP, and NO_3_–N could be attributed to the fact that these sampling sites were located near the river mouth, which discharges water into the lakes from surrounding agricultural farms (Xiao & Zang, 2010; Xiao et al., 2014). The indicator species for Group I were Chironomidae and *Corbicula fluminea*, which are relatively tolerant to eutrophic conditions (Gong et al., 2001). *C*. *fluminea* has also been shown to tolerate low temperatures (Gerard et al., 2009; Liu & Xiong, 2008). Group II, which mainly included sampling sites in Habuta Lake, was characterized by high COD_Mn_, TP, NH_3_–N, and NO_3_–N. Interestingly, the abundance of annelids such as *B*. *sowerbyi* and *Herpobdella* sp., which are associated with excess lake nutrients (Cai et al., 2017; Du et al., 2021), was remarkably high in Group II. This implies that, despite being located far away from the other lakes, the water sources of Habuta Lake are the same as those of the other lakes. Habuta Lake could be in a state of degradation owing to eutrophication.

Significant variability of environmental variables was recorded in Group IV, which encompassed sampling sites from the eastern part of Lianhuan Lake. COD_Mn_, TP, and NH_3_–N were markedly high, while DO was notably low, likely because of the dominance of human activities such as crop farming east of the lake (Xiao et al., 2014). Increased agricultural and urban land use can significantly change the physicochemical characteristics of freshwater systems (Huang et al., 2014; Johnson et al., 2007; Mathur et al., 2008). Such increases have been shown to increase nutrients and change macroinvertebrate compositions (Johnson et al., 1993; Kubosova et al., 2010; Yu et al., 2016). *G*. *pervia*, *Clinotarypus* sp., and *Tanypus* sp. were grouped into Group IV, possibly because they are tolerant of high levels of pollution (Wang, 2003). These species respond to organic pollution by increasing their abundance and their prevalence in Group IV supports this fact. They can live in extremely polluted waters with very low oxygen levels (Uwadiae, 2016).

This study also revealed that Delong Lake, Yangcaohao Lake, and Beiqin Lake, clustered in Group V, were characterized by good water quality, despite them being located in the upper reaches of Lianhuan Lake and sampled during summer when large amounts of surface runoff is discharged. This clearly demonstrates spatial variation in water quality. The fact that many indicator species were identified in Group V also implies that the conditions allowed many organisms to thrive.

Macroinvertebrates are an important part of the lake ecosystem, and their community structure characteristics are related to lake environmental variables. This study revealed that pH, TP, NO_3_–N, WT, DO, COND, COD_Mn_, and NH_3_–N had a significant effect on the classification of macroinvertebrate communities (Figure 5). pH is known to influence the composition and abundance of macroinvertebrate communities. A study by Tamiru (2019) indicated that alkaline water reduces macroinvertebrate abundance, biomass, and diversity. Moreover, tolerance studies have revealed that tolerance to pH varies between macroinvertebrate species (Ormerod et al., 1987). Based on the RDA and Spearman correlation analysis, the biomass of the macroinvertebrates, especially that of mollusks, was significantly negatively correlated with pH in this lake. This could be attributed to the high pH values (8–10) which are experienced in the lake year‐round (Li et al., 2009). Extreme pH environments can have direct toxic effects on mollusks and, under certain conditions, endanger the normal survival of various organisms (Wu et al., 2018). Peiffer et al. (1997) also noted that extreme pH conditions not only directly affect the birth rate of benthic invertebrates, reducing their biodiversity, but also cause benthic invertebrate poisoning by triggering the release of heavy metals. This could be another possible explanation for the negative influence of pH on macroinvertebrates in this study, as Li et al. (2009) revealed that the acidity and alkalinity of Lianhuan Lake changed with the differential enrichment of heavy metals caused by the discharge of industrial sewage.

Water temperature (WT) affects the physiological processes of organisms; thus, temperature dynamics may change their life cycle patterns and trophic interactions (Li et al., 2012). This may alter the community composition and biodiversity. Interestingly, there was no significant difference in the WT (*p* > .05) among the lakes (Table 1). However, the RDA results indicated that WT had a significant influence (*p* < .05) on macroinvertebrate species composition. This indicates that the WT affected the macroinvertebrates of Lianhuan Lake seasonally, rather than spatially. According to the RDA results, most of the macroinvertebrates were significantly positively correlated with WT, which is in agreement with other studies (Buss et al., 2004). Water temperature (WT) is an important factor in the embryonic development, larval growth, emergence, metabolism, and survival of macroinvertebrates (Haidekker & Hering, 2008). The fact that many indicator species were recorded in Group V further demonstrates that WT is an important factor, because these sites were sampled during summer and spring.

Nutrients are essential for maintaining the structure and function of lake ecosystems. However, excessive nutrients can reduce the water quality and deplete DO, leading to the death of aquatic organisms (Ouyang et al., 2018). On average, the macroinvertebrate species richness and abundance in this study exhibited a stress relationship with nutrients, mainly TP, Chla, and NO_2_–N. This result is consistent with the conclusion that high nutrient concentrations negatively affect benthic invertebrate species richness and abundance. This conclusion has been previously drawn in manipulative experiments and observational studies (Dodson et al., 2000; Wang et al., 2007). When the nutrients in a lake are excessive, as observed in Group IV, the water quality deterioration and DO depletion caused by the decomposition of algal bloom biomass is likely to reduce species richness (Wang et al., 2007), leading to a negative relationship between nutrients and species richness. The high nutrient concentration recorded in this lake could be attributed to the use of fertilizers to increase agricultural production.

## 5 CONCLUSION

In this study, the analysis of macroinvertebrate assemblages identified a gradient of macroinvertebrate diversity in Lianhuan Lake. It also captured the spatiotemporal variation in the macroinvertebrate community structure and identified the indicator species in the lake. The SOM analysis of the macroinvertebrate communities revealed that eutrophication has a serious impact on them. The differences in the community structure and environmental variables between Groups I and V were remarkable, and the indicator species reflected the environmental characteristics of each group of communities. The increasing alkalinity and eutrophication of the lake may have a serious impact on the macroinvertebrate community. This is clearly demonstrated by the significant negative correlation between macroinvertebrate biomass and pH, as well as the negative correlations between species richness and Shannon's diversity and TP. High‐intensity human disturbances, such as industrial and agricultural runoff, negatively impact the ecological environment and affect macroinvertebrate community structure. Thus, the macroinvertebrate community structure in Lianhuan Lake should be strengthened by improving the ecological environment and controlling environmental pollution (nonpoint source pollution) in the watershed. The impact of environmental variables on macroinvertebrates in lakes is a long‐term accumulating process. This study only spanned three seasons and did not cover all the lakes of Lianhuan Lake. Consequently, this study is subject to some time and space limitations. Watershed land‐use intensity is also a key factor affecting macroinvertebrates. Therefore, future research should focus on the impact of land use and other anthropogenic stressors on macroinvertebrates or compare future results to those of this study to explore the succession of the macroinvertebrate communities in Lianhuan Lake.

## CONFLICT OF INTEREST

The authors declare no conflict of interest.

## AUTHOR CONTRIBUTIONS

**Qianming Dou:** Conceptualization (equal); Data curation (equal); Formal analysis (equal); Investigation (equal); Methodology (equal); Software (equal); Supervision (equal); Visualization (equal); Writing – original draft (equal). **Xue Du:** Conceptualization (equal); Formal analysis (equal); Investigation (equal); Methodology (equal); Project administration (equal); Software (equal); Supervision (equal). **Yanfeng Cong:** Investigation (equal); Resources (equal); Supervision (equal). **Le Wang:** Investigation (equal); Writing – review & editing (equal). **Chen Zhao:** Investigation (equal); Methodology (equal); Resources (equal); Writing – review & editing (equal). **Dan Song:** Investigation (equal); Methodology (equal); Writing – review & editing (equal). **Hui Liu:** Investigation (equal). **Tangbin Huo:** Conceptualization (equal); Funding acquisition (equal); Investigation (equal); Methodology (equal); Project administration (equal); Resources (equal); Writing – review & editing (equal).

## Supporting information

Appendix S1

Appendix S2

## Data Availability

Macroinvertebrate and environmental variable data were deposited in the FigShare Digital Repository, https://doi.org/10.6084/m9.figshare.16577390.v3. Other relevant data were sourced from publicly accessible repositories or manuscripts.
