# Supplementary material for: Influence of environmental variables on macroinvertebrate community structure in Lianhuan Lake
Source: Ecol Evol. 2022 Feb 14;12(2):e8553. doi: 10.1002/ece3.8553 (PMC8843771; doi:10.1002/ece3.8553)
Supplement: Supplementary file 1 — Appendix S1 [file ECE3-12-e8553-s002.docx]

Quantitative error and structural error corresponding to different topological structures of self-organizing map (SOM)

| SOM topology structure | （20）  4*5 | （36）  6*6 | （42）  6*7 | （49）  7*7 | （72）  8*9 |
| --- | --- | --- | --- | --- | --- |
| quantization error | 1.084 | 0.996 | 0.973 | 0.938 | 0.928 |
| topographic error | 0.054 | 0.014 | 0.014 | 0.000 | 0.014 |
