# Supplementary material for: Influence of environmental variables on macroinvertebrate community structure in Lianhuan Lake
Source: Ecol Evol. 2022 Feb 14;12(2):e8553. doi: 10.1002/ece3.8553 (PMC8843771; doi:10.1002/ece3.8553)
Supplement: Supplementary file 2 — Appendix S2 [file ECE3-12-e8553-s001.docx]

List of macroinvertebrates in Lianhuan Lake and their distribution based on self-organizing map (SOM) grouping. Species with higher-than-average abundance are highlighted in bold

| **Order** | **Family** | **Species** | **Abbreviation** | **I** | **II** | **III** | **IV** | **V** |
| --- | --- | --- | --- | --- | --- | --- | --- | --- |
| Ephemeroptera | Ephemeridae | *Ephemera* sp. | Ephem | **+** |  | **+** |  |  |
| Hemiptera | Corixidae | *Mirconecta* sp.1 | Mirc1 |  | **+** |  |  |  |
|  |  | *Mirconecta* sp.2 | Mirc2 |  | **+** |  |  |  |
|  |  | *Sigara* sp.1 | Siga1 |  | **+** |  |  |  |
|  |  | *Sigara* sp.2 | Siga2 |  | **+** |  |  |  |
| Diptera | Ceratopogonidae | ***Culicoides* sp.** | Culic | **+** | **+** | **+** | **+** |  |
|  | Culicidae | *Chaoborus* sp. | Chaob | **+** |  | **+** |  | **+** |
|  | Chironomidae | ***Conchapelopia* sp.1** | Conc1 | **+** | **+** | **+** |  | **+** |
|  |  | *Conchapelopia* sp.2 | Conc2 |  |  | **+** | **+** |  |
|  |  | ***Tanytarsus* sp.1** | Tany1 |  | **+** | **+** | **+** | **+** |
|  |  | *Tanytarsus* sp.2 | Tany2 | **+** | **+** |  |  |  |
|  |  | ***Procladius* sp.1** | Proc1 | **+** |  | **+** | **+** | **+** |
|  |  | ***Procladius* sp.2** | Proc2 |  | **+** | **+** |  | **+** |
|  |  | ***Chironomus* sp.** | Chiro |  |  |  | **+** | **+** |
|  |  | *Einfeldia* sp. | Einfe | **+** | **+** |  | **+** |  |
|  |  | ***Anatopynia* sp.** | Anato | **+** |  | **+** |  | **+** |
|  |  | *Clinotarypus* sp. | Clino |  |  |  | **+** |  |
|  |  | *Tanypus* sp. | Tanyp |  |  |  | **+** | **+** |
|  |  | *Polypedilum* sp. | Polyp | **+** |  | **+** |  |  |
|  |  | *Acricotopus* sp. | Acric | **+** |  |  | **+** |  |
|  |  | *Demicryptochironomus* sp. | Demic |  |  | **+** |  |  |
|  |  | *Cricotopus* sp. | Crico |  |  |  |  | **+** |
| Odonata | Cordulegasteridae | *Anotogaster* sp. | Anoto |  | **+** |  |  |  |
| Basommatophora | Planorbidae | ***Gyraulus albus*** | Gyrau |  |  | **+** | **+** | **+** |
|  | Lymnaeidae | ***Radix pereger*** | Raper |  | **+** | **+** | **+** | **+** |
|  |  | *Radix ovata* | Raova |  | **+** | **+** | **+** | **+** |
|  |  | *Radix lagotis* | Ralag |  |  |  |  | **+** |
|  |  | ***Radix auricularia*** | Raaur |  | **+** | **+** |  | **+** |
|  |  | ***Galaba* sp.** | Galab |  | **+** |  |  | **+** |
|  |  | ***Galba pervia*** | Gaper |  |  | **+** | **+** |  |
|  | Valvatidae | ***Valvata piscinalis*** | Vapis |  | **+** | **+** |  |  |
| Mesogastropoda | Stenothyridae | ***Stenothyra glabra*** | Stgla |  | **+** |  |  | **+** |
|  | Bithyniidae | ***Parafossarulus striatulus*** | Pastr |  | **+** |  | **+** | **+** |
| Veneroida | Corbiculidae | *Corbicula fluminea* | Coflu | **+** |  |  |  |  |
|  | Sphaeriidae | *Sphaerium lacustre* | Splac |  | **+** | **+** |  |  |
| Unionoida | Unionidae | *Unio douglasiae* | Undou |  | **+** |  |  | **+** |
|  |  | *Anodonta woodiana* | Anwoo |  |  |  |  | **+** |
| Rhynchobdellida | Glossiphonidae | *Glossiphonia* sp. | Gloss |  | **+** |  |  |  |
| Pharyngobdellida | Herpobdellidae | *Herpobdella* sp. | Herpo | **+** | **+** |  |  | **+** |
| Oligochaeta plesiopora | Tubificidae | ***Branchiura sowerbyi*** | Brsow | **+** | **+** |  |  |  |
|  |  | *Limnodrilus* sp.1 | Limn1 |  |  | **+** |  | **+** |
|  |  | *Limnodrilus* sp.2 | Limn2 |  |  | **+** |  | **+** |
|  |  | *Limnodrilus* sp.3 | Limn3 |  | **+** |  | **+** |  |
| Decapoda | Palaemonidae | *Exopalaemon modestus* | Exmod |  | **+** |  |  |  |
